# Multimodal Imaging of Cancer Therapy-Related Cardiac Dysfunction in Breast Cancer—A State-of-the-Art Review

**DOI:** 10.3390/jcm12196295

**Published:** 2023-09-29

**Authors:** Michael Cronin, Mehreen Seher, Shahram Arsang-Jang, Aoife Lowery, Michael Kerin, William Wijns, Osama Soliman

**Affiliations:** 1 CORRIB Core Laboratory, University of Galway, H91 TK33 Galway, Irelandm.--2@nuigalway.ie (M.S.);; 2 Precision Cardio-Oncology Research Enterprise (P-CORE), H91 TK33 Galway, Ireland; 3 CURAM Centre for Medical Devices, H91 TK33 Galway, Ireland; 4 Discipline of Surgery, Lambe Institute for Translational Research, University of Galway, H91 TK33 Galway, Ireland; 5 Discipline of Cardiology, Saolta Group, Galway University Hospital, Health Service Executive and CORRIB Core Laboratory, National University of Ireland Galway (NUIG), H91 TK33 Galway, Ireland

**Keywords:** multimodal imaging, cancer therapy-related cardiac dysfunction, echocardiography, cardiac magnetic resonance imaging, nuclear imaging, cardiac computed tomography, global longitudinal strain, left ventricular ejection fraction

## Abstract

Background: This review focuses on multimodality imaging of cardiotoxicity in cancer patients, with the aim of evaluating the effectiveness of different techniques in detecting and monitoring cardiac changes associated with cancer therapy. Methods: Eight studies were included in the review, covering various imaging modalities such as cardiac magnetic resonance imaging, echocardiography, and multigated acquisition scanning. Results: Cardiac magnetic resonance imaging emerged as the most definitive modality, offering real-time detection, comprehensive assessment of cardiac function, the ability to detect early myocardial changes, and superior detection of cardiotoxicity when compared to the other imaging modalities. The studies also emphasize the importance of parameters such as left ventricular ejection fraction and global longitudinal strain in assessing cardiac function and predicting cardiotoxicity. Conclusion: Due to the common use of HER2 agents and anthracyclines within the breast cancer population, the LVEF as a critical prognostic measurement for assessing heart health and estimating the severity of left-sided cardiac malfunction is a commonly used endpoint. CTRCD rates differed between imaging modalities, with cardiac MRI the most sensitive. The use of multimodal cardiac imaging remains a nuanced area, influenced by local availability, the clinical question at hand, body habits, and medical comorbidities. All of the imaging modalities listed have a role to play in current care; however, focus should be given to increasing the provision of cardiac MRI for breast cancer patients in the future to optimize the detection of CTRCD and patient outcomes thereafter.

## 1. Introduction

We present a state-of-the-art review of multimodal imaging use within the breast cancer population. The scope of this systematic review is to comprehensively evaluate the existing literature on the use of different cardiovascular imaging modalities and imaging markers in the detection of cardiotoxicity induced by anti-cancer treatments. The primary objective is to determine which imaging modality is most effective in identifying cardiotoxicity. The review will also address secondary objectives such as assessing the accuracy, reliability, sensitivity, and specificity of different imaging modalities and markers, as well as the advantages and limitations of each method, particularly when subgroups of patients are involved.

### 1.1. Cardiotoxicity

Cardiotoxicity is a significant side effect of many cancer treatments, including chemotherapy and radiotherapy. It can lead to damage to cardiomyocytes, causing various cardiac dysfunctions, such as left ventricular dysfunction, myocardial infarction, arrhythmias, and heart failure [1]. Chemotherapeutic agents, such as anthracyclines, fluoropyrimidines, and cyclophosphamide, can induce damage to the myocardium, leading to heart failure, arrhythmias, and other cardiovascular complications [2]. Therefore, the timely detection and monitoring of cardiotoxicity are essential for the safe and effective management of cancer patients.

### 1.2. HER2 Agents

Trastuzumab is a monoclonal antibody used in the treatment of HER2-positive breast cancer, with its relationship to cardiotoxicity previously documented [3]. The mechanism of trastuzumab-induced cardiotoxicity is yet to have an international consensus but is thought to be due to the inhibition of HER2 signaling in cardiomyocytes, which can lead to impaired cardiac function and heart failure [4], via an increase in reactive oxygen species in cardiomyocytes [5], and also a genetic predisposition via the EYS gene [6]. Previous publications have found this incidence of symptomatic heart failure to be 6.6% [7,8] in populations of 61 and 244, respectively. This risk of cancer therapy-related cardiotoxicity is increased by the additional use of anthracyclines [9,10]. Reassuringly, only a small percentage of the cardiotoxicity experienced due to trastuzumab therapy is permanent [11], with recovery in most cases.

### 1.3. Tyrosine Kinase Inhibitors 

Tyrosine kinase inhibitors (TKIs) are a class of targeted cancer therapies that work by inhibiting the signals that encourage cancer development. They have a recognized relationship with adverse cardiac events [12,13], with TKI-induced cardiotoxicity related to an increased risk of cardiovascular events and death in cancer patients [14]. Similar to trastuzumab, TKI-induced cardiotoxicity is reversible in most cases [15]. The mechanism of cardiovascular toxicity has been described as secondary to “off-target” effects and varies amongst agents [16,17].

### 1.4. Anthracyclines

One of the most common causes of cardiotoxicity is the use of anthracycline chemotherapy drugs. These drugs have been shown to induce oxidative stress and DNA damage in cardiac cells, leading to cell death and impaired cardiac function [18,19]. Additional descriptions have been the induction of inflammation in cardiac tissue, leading to fibrosis and impaired cardiac function [20], and the presence of genes responsible for ATP binding cassettes, nitric oxide synthase, and alcohol dehydrogenase [21]. The extent of anthracycline-induced cardiotoxicity carries a linear relationship with agent dosage [22].

### 1.5. Alkylating Agents

Alkylating agents are used in the treatment of various cancers (including breast, lymphoma, and leukemia) and can induce cardiotoxicity through multiple mechanisms, including myocardial depression and vasospasm [23]. The reported incidence of CTRCD has been as high as 10.2% [24].

### 1.6. Radiotherapy

In addition to chemotherapy drugs, radiation therapy can also cause cardiotoxicity. Various factors have been associated with the mechanism of cardiotoxicity, including oxidative stress, deoxyribonucleic acid degradation, and inflammatory cytokine release with subsequent fibrotic changes of the myocardium, pericardium, and valvular structures, as well as the vasculature [25]. The incidence of radiation-induced cardiotoxicity varies depending on the dose and volume of the heart irradiated, as well as the patient’s baseline cardiac function [26]. A cumulative dose of 5–15 Gy is considered moderate risk, and >15 Gy is considered high risk [27], particularly when a volume inclusive of the mediastinum is involved. Early detection and diagnosis of cardiotoxicity are crucial to improving patient outcomes. However, cardiotoxicity can be challenging to diagnose, as symptoms may not be evident until cardiac dysfunction has become severe. Furthermore, the symptoms of cardiotoxicity may overlap with those of cancer or other co-morbidities, making it difficult to attribute them solely to cardiotoxicity [28].

### 1.7. Multimodal Imaging

Multimodality imaging is effective in the early detection and monitoring of cardiotoxicity in cancer patients. Previous publications [29] demonstrate that the combination of echocardiography and cardiac MRI provides a more accurate assessment of left ventricular function than either modality alone. However, despite the potential benefits of multimodality imaging, there are some limitations and challenges to its use in clinical practice. These include the cost of imaging, the need for specialized training and expertise to interpret results, and the lack of standardized protocols for imaging and interpretation [28].

### 1.8. Echocardiography

Echocardiography is a widely used imaging modality for the assessment of cardiac function. It is non-invasive, portable, relatively inexpensive, and does not use ionizing radiation. Echocardiography via the use of global longitudinal strain (GLS) can provide information on left ventricular ejection fraction (LVEF), regional wall motion abnormalities (RWMA), and valvular function [30]. It is particularly useful in the early detection of cardiotoxicity, when changes in LVEF can be detected before the onset of symptoms [1,31,32,33], with three-dimensional image acquisition preferable due to reduced intra-observer variability [34,35]. A change in LVEF of ≥10 percentage points or a final EF of <53% following high-dose chemotherapy with autologous stem cell transplantation was associated with a higher incidence of cardiac events [31]. A reduction in GLS of at least 15% following anthracycline treatment established a correlation between an increase in the risk of cardiac events, such as heart failure, and cardiovascular death [29]. Further, an elevated risk of later cardiotoxicity was linked to a drop in GLS of at least 2.5% within the first year after anthracycline treatment [28], and a decrease in GLS of ≥10% following treatment with immune checkpoint inhibitors was associated with an increased risk of cardiac events [34]. Global circumferential strain (GCS) is a measure of the myocardial deformation around the left ventricle in a circumferential direction during systole that can be obtained using cardiovascular imaging modalities such as echocardiography and cardiac MRI; however, preliminary studies have not proved clinical benefit [36]. Tissue doppler imaging (TDI) via echocardiography can establish early subtle changes of diastolic impairment treated with anthracyclines [37], with isovolumetric relaxation time and the evaluation standard within most accredited examinations, as well as deceleration time and E and A wave assessments [38]. Within the limitations of echocardiography are limited sensitivity and reproducibility, particularly for the detection of subtle changes in myocardial function [39], as well as a dependence on patient body habits and adequate imaging windows. 

### 1.9. Magnetic Resonance Imaging

Magnetic resonance imaging (MRI) is a versatile imaging modality that can provide information on both cardiac function and structure. It can detect RWMA, changes in LVEF, and myocardial fibrosis. Myocardial fibrosis is a common finding in patients with cardiotoxicity. It is a marker of irreversible damage to the heart muscle and can be detected by late gadolinium enhancement on MRI [40], with an increased risk of subsequent heart failure in patients undergoing chemotherapy [41]. Changes in GLS on MRI are associated with an increased risk of CTRCD [42], with the extent of late gadolinium enhancement being a marker of the severity of CTRCD [22]. Previous publications have described its superiority to echocardiography in the detection of (sub)clinical CTRCD, specifically in lymphoma treated with anthracyclines [43], anthracycline use in breast cancer [22], and also its role in the diagnosis of checkpoint inhibitor therapy-induced myocarditis [44]. Myocarditic changes secondary to immune checkpoint inhibitors constitute a major diagnostic criterion [27] and are defined by the 2018 updated Lake Louise criteria [45], which describe specific features seen during T1-weighted and T2-weighted imaging acquisition. MRI is now recognized for its superior diagnostic accuracy compared to other imaging modalities, such as echocardiography and nuclear medicine imaging. However, the high cost, longer scanning time, dependence on renal function for the use of gadolinium, and lower availability of cardiac MRI in some settings limit its widespread use.

### 1.10. Cardiac Computed Tomography

In recent years, cardiac computed tomography angiography (CCTA) has emerged as a valuable tool in the diagnosis and management of various cardiovascular diseases, including coronary artery disease and coronary anomalies [46], where a significant coronary artery stenosis is taken to be >70%. It carries a high spatial resolution, which allows for the detection of small changes in cardiac morphology and function [46]. CCTA utilizes an ECG-gated 64-slice CT scanner, with various image acquisition protocols recognized [47]. It has also been shown to be useful in the assessment of cardiomyopathy. CCTA-derived extra-cellular volume fraction followed similar dynamics when compared to LVEF and GLS measured by TTE in a breast cancer population treated with anthracyclines [48]. A major limitation is the use of ionizing radiation and contrast agents that can pose risks, particularly in patients with renal impairment [49].

### 1.11. Nuclear Imaging

Nuclear cardiac scanning is a non-invasive imaging technique that utilizes radiotracers to evaluate myocardial perfusion and function. It is an alternative imaging modality in patients who cannot undergo other imaging modalities (e.g., MRI), with multigated radionuclide angiography (MUGA) and single-photon emission computed tomography (SPECT) further acquisition methods that, via a range of radioisotopes, can assess LVEF and for perfusion defects [50]. Prior examples of its clinical use include PET imaging with fluorine-18-labeled deoxyglucose (FDG) in identifying cardiotoxicity in lymphoma patients treated with anthracyclines [51]. In this case, myocardial glucose uptake was considerably lower in those who suffered cardiotoxicity than in those who did not. The use of radiation and radiotracers within nuclear imaging can pose risks, particularly in pregnant women and patients with renal impairment, which poses some limitations to its use. If a dual-phase study is needed, this can be quite laborious and not feasible for a patient who is traveling a distance to undergo the test.

### 1.12. Cardiac Biomarkers

Cardiac biomarkers have some relevance in the screening of the cardiotoxicity of anti-cancer treatments, including HER2 therapies [52]. To this end, they are included in the European guidelines for baseline assessment, recognition of CTRCD, and monitoring of recovery [27]. However, there is a definite consensus in their clinical relevance as the situation varies between patient population, primary tumor type, and anti-cancer agent. Some of the difficulty arising in this area is the fact that studies publish limited population numbers who have suffered cardiotoxicity, with 15, 3, 24, and 11 patients, respectively, having suffered cardiotoxicity in publications that did not find any predictable association between biomarkers and trastuzumab [53,54,55,56]. Previous meta-analyses [57] found that in a population treated with anti-HER2 agents with/without anthracycline, elevated troponin carried a higher risk of left ventricular systolic dysfunction, but this pattern was not consistently associated with natriuretic peptides (10 studies, 462 patients). The literature remains varied in recommendation, and ultimately biomarkers may/may not play a leading role in patient evaulation, but cardiac troponin and natriuretic peptides give some clinical information that should be taken into consideration in the context of patient presentation and other diagnostics at hand.

## 2. Materials and Methods

For published articles, the following electronic databases were utilized: PubMed/MEDLINE, Embase, Cochrane Central Register of Controlled Trials (CENTRAL), and Web of Science. The search strategy combined keywords associated with the following ideas with medical subject headings (MeSH) terms: echocardiogram, single-photon emission computed tomography (SPECT), MRI, CT, strain imaging, contrast agents, perfusion imaging, and cancer treatment-related cardiotoxicity. The search strategy included relevant synonyms and related terms and used Boolean operators (AND, OR) to combine the different search concepts.

In addition to the electronic database search, the following sources of unpublished or gray literature were also searched: the World Health Organization, the International Clinical Trials Registry Platform (WHO ICTRP), the conference proceedings of relevant cardiology and radiology conferences, and ClinicalTrials.gov. To find additional studies, the reference lists of the included studies and pertinent systematic reviews were also hand-searched.

Duplicate results were eliminated after importing all search results into a reference management program. The surviving studies’ titles and abstracts were reviewed by two impartial reviewers to determine their eligibility based on the requirements for inclusion and exclusion criteria. Studies that satisfied the eligibility requirements had their full-text papers retrieved, and two independent reviewers decided whether to include those studies in the systematic review. A third reviewer or a majority vote was used to settle any disputes.

The inclusion and exclusion criteria were consistently applied to all studies. The inclusion criteria were as follows: Randomized controlled studies, prospective and observational studies, studies presented in English, patient populations who had a history of breast cancer treatment and underwent at least two of the following cardiovascular imaging modalities: echocardiography, CT, MRI, multigated acquisition (MUGA) scanning, and were 18 years or older. Table 1 provides the information extracted from each included study, which was taken from text, tables, and images. The exclusion criteria were as follows: Studies that did not fit the requirements for eligibility, studies that did not report outcomes related to cancer treatment-related cardiotoxicity, studies published as conference abstracts or posters only, and studies published before the year 2000. The risk of bias and reporting transparency of the included studies was assessed using the PROBAST tool [58]. 

In the participant selection domain of the PROBAST model, the signaling questions addressed issues such as the representativeness of the study sample and the handling of missing data. In the predictor domain, the signaling questions focused on issues such as the measurement of predictors and the handling of missing data. In the outcome domain, the signaling questions assessed the quality and completeness of outcome data. Finally, in the analysis domain, the signaling questions addressed issues such as model overfitting and handling of missing data.

In addition to the PROBAST tool, reporting transparency was also assessed using the Transparent Reporting of a Multivariable Prediction Model for Individual Prognosis or Diagnosis (TRIPOD) statement [59]. The TRIPOD statement provides guidelines for reporting multivariable prediction model studies. It comprises a 22-item checklist that includes items related to the study design, participant selection, predictor variables, outcome variables, and statistical analysis.

Overall, the risk of bias and reporting transparency of the included studies were found to be variable. Some studies were found to have a high risk of bias in one or more domains, while others had a low risk of bias. Similarly, some studies were found to have incomplete or unclear reporting, while others had more transparent reporting. These findings highlight the need for standardized reporting and better adherence to reporting guidelines in prediction model studies.

## 3. Results

**Figure 1:**
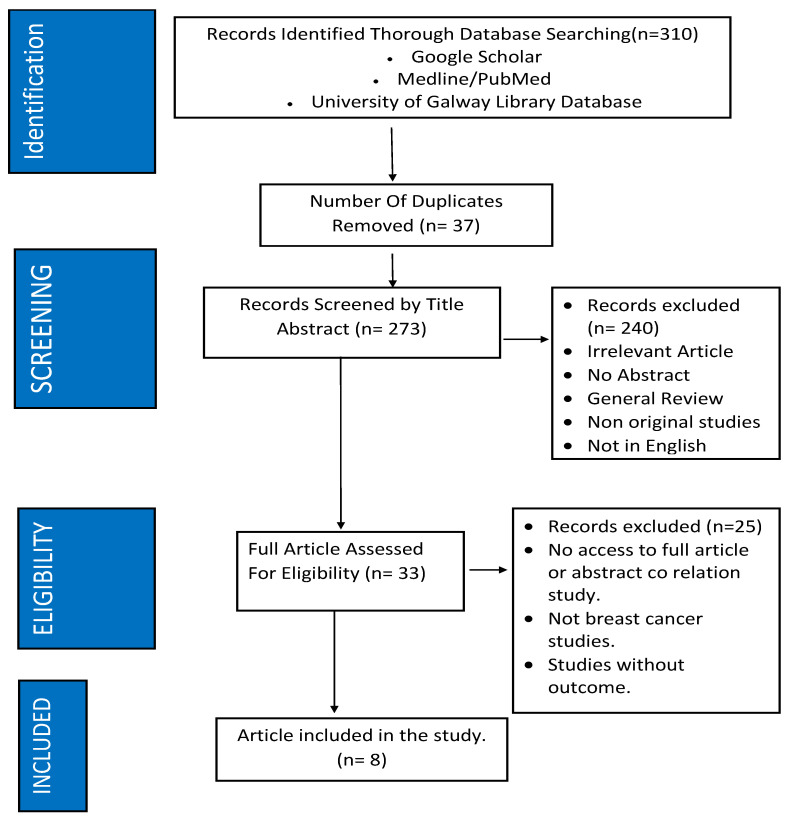
Methodology of study inclusion.

Figure 1 demonstrates the eligibility process in the literature review. Ultimately, in this review, eight studies focusing on modalities used in detecting cancer-caused cardiotoxicity met eligibility criteria [60,61,62,63,64,65,66,67], with all eight populations having breast cancer from stages one to three. The characteristics of these papers can be found in Table 2, with a summary of study outcomes in Table 3. Several common findings emerged from these studies, highlighting the importance of early detection and monitoring of cardiotoxic effects in patients undergoing chemotherapy or targeted therapy.

The literature highlighted in this review demonstrates the use of different cardiovascular imaging modalities predominantly within the realm of HER2 therapies, anthracyclines, and alkylating agents. The investigation of HER2 patients for cardiotoxicity is relevant to their prognosis, and although HER2 status in invasive cancer is established in a patient’s prognosis, its presence in ductal carcinoma in situ (DCIS) is less so, with current guidance suggesting against HER2 testing in these patients [68]. As the emphasis for CTRCD generally rests around symptomatic heart failure or asymptomatic LV systolic dysfunction [27], there is a predominance within the imaging papers selected regarding ejection fraction. Immune checkpoint inhibitors, TKIs, vascular endothelial growth factor inhibitors, RAF/MEK inhibitors, androgen-deprivation therapies, endocrine therapies, and radiotherapy were also not included within the studies, and this was reflected in the choice of cardiovascular imaging and the clinical question that required answering. 

The selected studies report several advantages for cardiac MRI in the assessment of cardiotoxicity, suggesting it provides high spatial resolution and excellent tissue characterization, allowing for the detection of early myocardial changes that may not be picked up by TTE and MUGA imaging [66]. Houbois et al. [64] found using cardiac MRI that 28% of patients developed CTRCD, as opposed to 22% by 2D echocardiography within the same population. It enables the assessment of ventricular function, including LVEF and GLS, which have shown promise in the early detection of cardiac dysfunction [60], while also providing an assessment of valvular function [65]. Terui Y., et al. [66] also found that the native T1 values were an independent predictive factor for the development of CTRCD. It is a non-invasive imaging modality that does not involve ionizing radiation, making it safe for repeated use in patients undergoing cancer therapy. Several studies have demonstrated the feasibility of CMR in detecting cardiotoxic effects in breast cancer patients receiving anthracycline-based chemotherapy or targeted therapies [62,64].

Depending on the clinical question at hand, each of the named imaging modalities has a use in different situations and is highly dependent on availability and local expertise. The use of strain imaging techniques, such as tissue velocity and strain imaging, showed promise in predicting early left ventricular dysfunction. Fallah-Rad et al. [61] reported that cardiac biomarkers, tissue velocity, strain imaging, and MRI imaging could aid in predicting left ventricular dysfunction in breast cancer patients receiving trastuzumab therapy. Echocardiography compared favorably with MRI in strain assessment, with Houbois et al. [64] comparing serial cardiac MRI strain measurements with echocardiography and finding comparable results, suggesting that echocardiography could be a reliable alternative for identifying cardiotoxicity in breast cancer patients. In the studies including MUGA imaging [63,65,67], its use was pertaining to LVEF measurements without reference to perfusion defect assessment.

A common measure of cardiotoxicity using the LVEF parameter (although there are others) is a decrease in absolute value to below 50% without a decrease from the baseline of >10% [27]. However, certain medications demonstrated potential for preventing or mitigating this cardiotoxic effect. Guglin et al. [62] found that both lisinopril and carvedilol could prevent trastuzumab cardiotoxicity in patients with breast cancer. This study found that the average change in mean LVEF in the group administered Carvedilol was −4.5 ± 0.8% and in the group administered Lisinopril was −4.0 ± 0.8%. 

## 4. Discussion

Our study provides a state-of-the-art review of studies focused on multimodality imaging of cardiotoxicity, showing that choice of imaging is dependent on the anti-cancer therapy agent, clinical question, and local availability/expertise. Ultimately, this literature is of most relevance to breast cancer patients receiving HER2 agents, anthracyclines, and alkylating agents, where the CTRCD of note is cardiomyopathy, witnessed as a depressed LVEF/GLS. By including a diverse range of studies from a number of countries, the review offers a comprehensive analysis of the topic, enhancing the reliability and validity of the findings. Furthermore, given that this review focuses on studies conducted on patients in the real world undergoing cancer treatment, we feel this makes the findings directly applicable to clinical practice. By considering the implications of the imaging modalities in real-world scenarios, the study enhances its practical significance and relevance to healthcare professionals.

Each of TTE, MUGA, and cardiac MRI have a role in these patients care. Systems of healthcare, equipment availability, private/public funding, and the availability of local expertise all determine imaging choice. The breast cancer population, by virtue of this background, has a multimodal approach, with a predominance of different modalities used across different jurisdictions. Indeed, cardiac CT is not included in our review but is well-established in the detection of coronary artery disease that may have been accelerated by radiotherapy [69]. The use of MRI provides a gold standard modality for detecting cardiotoxicity, particularly early/subclinical findings, and current literature suggests that incorporating it into routine clinical practice could enhance the early detection and monitoring of cardiac dysfunction in cancer patients. Clinicians should consider including MRI as a part of the baseline assessment before initiating cardiotoxic therapies in specific high-risk populations, defined as high-risk by their medical history or anti-cancer treatment, and serial MRI imaging could be utilized to monitor changes in cardiac structure and function over time. This proactive approach could facilitate early intervention and optimize patient outcomes. We do not endorse alternating between different imaging modalities during treatment or follow-up [70]. 

Our review was limited by the heterogeneity of the studies. The included studies varied in terms of sample size, study design, patient population, and imaging protocols. This heterogeneity could introduce potential sources of bias and limit the ability to draw definitive conclusions. There was a lack of standardized protocols and criteria for defining and assessing cardiotoxicity across different imaging modalities, which could potentially lead to inconsistencies and challenges in comparing results across studies. Longitudinal studies with extended follow-up periods would provide more robust evidence regarding the long-term outcomes of cardiotoxicity detection. We felt the studies included in the review may have inherent selection biases due to their specific inclusion and exclusion criteria. This could affect the generalizability of the findings to broader patient populations. 

Many of the included studies may have relatively short follow-up periods, and only one study [61] provided data regarding true and false positives/negatives, precluding statistical analysis across the studies. 

### Gaps in the Literature

One of the major gaps in the current literature regarding multimodality imaging is the lack of standardization in the use of established protocols for cardiotoxicity assessment and interpretation of imaging findings. However, international societies are making progress in this regard [27], and these recent protocols are yet to be the norm internationally. Different imaging modalities are effective in detecting cardiotoxicity, but there is a lack of consensus on the optimal imaging modality to use in specific patient populations, given what we have already mentioned regarding different health systems.

The use of multiple imaging modalities can be expensive, and there is a need to evaluate the cost-effectiveness of these modalities in different patient populations. Comparative studies that evaluate the costs and benefits of different imaging modalities could help guide clinical decision-making and resource allocation. While many studies have evaluated the utility of imaging modalities in detecting early cardiac dysfunction, there is a need for studies that evaluate the long-term prognostic value of these modalities in predicting adverse cardiovascular outcomes. Predictive models would enable clinicians to identify patients who are at high risk of developing cardiotoxicity, allowing for the implementation of preventive strategies. Individual imaging modalities are effective in detecting cardiotoxicity, but there is a need to evaluate the added value of combining different imaging modalities. Multimodality imaging approaches could provide a more comprehensive assessment of cardiac function and facilitate the early detection of cardiac dysfunction. A summary of the above can be found in Table 4.

## 5. Conclusions

The influence of breast cancer therapy on the heart, known as cardiotoxicity, is the topic of substantial investigation. Studies previously employed imaging modalities such as TTE, cardiac MRI, and MUGA to assess cardiotoxicity. Of particular relevance within the breast cancer population is the LVEF, a critical prognostic measurement for assessing heart health and estimating the severity of left-sided cardiac malfunction. CTRCD rates differed between imaging modalities, with cardiac MRI the most sensitive. The use of multimodal cardiac imaging is a nuanced area, influenced by local availability, the clinical question at hand, body habits, and medical comorbidities. All of the imaging modalities listed have a role to play in current care; however, focus should be given to increasing the provision of cardiac MRI for breast cancer patients in the future to optimize the detection of CTRCD and patient outcomes thereafter.

## Figures and Tables

**Table 1:** Information extracted from each study.

Categories	Data Extracted
Study Characteristics	- Author - Year of publication - Study design - Country of origin - Funding source
Participant Characteristics	- Sample size - Age - Sex - Type of cancer - Type of cancer treatment
Intervention and Character Details	- Type of cardiovascular imaging modality used - Imaging markers assessed - Duration and frequency of follow-up - Any co-interventions or medications used
Outcome Measures	- Primary and secondary outcomes reported - Method of outcome assessment - Time points of outcome assessment
Results	- Study findings for each outcome measure - Effect size - Confidence intervals - *p*-values

**Table 2:** Study characteristics.

Author(s)	Source of Data	No. of Subjects	Imaging Technique	Treatment
Boekhout et al., 2016	Type of study: Randomized clinical trial Place of study: The Netherlands Years of study: 2011–2015	206	TTE, MUGA	Trastuzumab
Fallah-Rad et al., 2011	Type of study: Observational study Place of study: Canada Years of study: 2011	42	Strain Imaging, CMR	Trastuzumab
Guglin et al., 2019	Type of study: Randomized controlled trial. Place of study: USA Years of study: 2019	468	TTE, MUGA	Trastuzumab Lisinopril Carvedilol Phosphate-Extended Release
Henry et al., 2018	Type of study: Observational study Place of study: USA Years of study: 2018	16,456	TTE, MUGA, CMR	Trastuzumab
Houbois et al., 2021	Type of study: Prospective observational study Place of study: Denmark Years of study: 2014–2018	125	CMR, Echo	Trastuzumab Anthracyclines
Kar et al., 2023	Type of study: Prospective study Place of study: USA Years of study: 2023	32	Stimulated Echoes (DENSE) MRI, TTE, GLS	Trastuzumab Doxorubicin Cyclophosphamide Taxol Carboplatin Pertuzumab
Terui et al., 2023	Type of study: Prospective observational study Place of study: Japan Years of study: 2016–2021	83	CMR, TTE	Trastuzumab Anthracyclines
Yu et al., 2020	Type of study: Prospective observational study Place of study: Japan Years of study: 2016–2021	53	TTE, MUGA, CMR	Trastuzumab

**Table 3:** Imaging modalities used in detecting cardiotoxicity following chemotherapy in breast cancer patients between stages 1 and 3.

Study	Title	Imaging Technique	Main Study Outcomes
Boekhout et al., 2016	Angiotensin-2 Reception Inhibition with Candesartan to prevent Trastuzumab-related Cardiotoxic events in patients with early Breast Cancer	TTE, MUGA	At least 1 of the 2 primary cardiac end points was manifested by 36/206 cases. There were 3.8% more cardiac events in the candesartan group than in the placebo group. The 2-year cumulative incidence of cardiac events was 0.28 (95% CI, 0.13–0.40) in the candesartan group and 0.16 (95% CI, 0.08–0.22) in the placebo group (*p* = 0.56). Candesartan did not affect changes in NT-pro-BNP and HS-TnT values, and these biomarkers were not associated with significant changes in LVEF
Fallah-Rad et al., 2011	The Utility of Cardiac Biomarkers, Tissue Velocity, Strain Imaging, and Cardiac MRI in predicting early LV Dysfunction in patients with HER2-positive Breast Cancer treated with adjuvant Trastuzumab therapy	TTE, MRI	Ten (24%) women developed trastuzumab-induced CM. Decreased LVEF at 12-month follow-up: by TTE from 61% +/− 9 to 49% +/− 4, and by CMR: from 66% +/− 5% to 47 +/− 4%
Guglin et al., 2019	Randomized Trial of Lisinopril vs. Carvedilol to prevent Trastuzumab Cardiotoxicity in patients with Breast Cancer	TTE, MUGA	CTRCD in 32% of patients on placebo, 29% on carvedilol (Anthracycline group HR 0.49 (*p* = 0.009), non-anthracycline HR 1.05 (*p* = 0.559)), 30% on lisinopril (Anthracycline group HR 0.53 (*p* = 0.015), and non-anthracycline group HR 1.17 (*p* = 0.689))
Henry et al., 2018	Cardiotoxicity and Cardiac Monitoring among Chemotherapy-treated Breast Cancer Patients	TTE, MUGA, MRI	A total of 692 patients (4.2%) developed HF after chemotherapy: 2.1% (<35 years old), 2.9% (36–49 years old), 3.5% (50–64 years old), and 8.3% (>65 years old)
Houbois et al., 2021	Serial Cardiovascular MR Strain Measurements to identify Cardiotoxicity in Breast Cancer	TTE, MRI	In total, 28% of patients developed CTRCD by CMR and 22% by 2DE. A 15% relative reduction in 2DE-GLS increased the CTRCD odds by 133% at subsequent follow-up, compared with 47%/50% by tagged-CMR GLS/GCS and 87% by FT-GCS
Kar et al., 2023	Can GLS with MR prognosticate early CTRCD in Breast Cancer Patients? A Prospective Study	TTE, MRI	GLS worsened from baseline to the 3- and 6-month follow-ups (−19.1 ± 2.1%, −16.0 ± 3.1%, −16.1 ± 3.0%; *p* < 0.001). Univariable Cox regression showed the 3-month GLS significantly associated as an agonist (hazard ratio [HR]-per-SD: 2.1; 95% CI: 1.4–3.1; *p* < 0.001) and LVEF as a protector (HR-per-SD: 0.8; 95% CI: 0.7–0.9; *p* = 0.001) for CTRCD occurrence. Bivariable regression showed the 3-month GLS (HR-per-SD: 2.0; 95% CI: 1.2–3.4; *p* = 0.01) as a CTRCD prognostic factor independent of other covariates, including LVEF (HR-per-SD: 1.0; 95% CI: 0.9–1.2; *p* = 0.9)
Terui et al., 2023	Usefulness of Cardiac MR for early detection of CTRCD in Breast Cancer Patients	TTE, CMR	In total, 8.4% of subjects developed CTRCD. LVEF and GLS were significantly decreased after chemotherapy (LVEF, from 71.2 ± 4.4 to 67.6 ± 5.8%; GLS, from −27.9 ± 3.9 to −24.7 ± 3.5%, respectively, both *p* < 0.01). The native T1 value was also significantly elevated after chemotherapy (from 1283 ± 36 to 1308 ± 39 msec, *p* < 0.01)
Yu et al., 2020	Cardiotoxicity Surveillance and risk of Heart Failure During HER2-targeted therapy	TTE, MUGA, MRI	In total, 14.7% of patients developed CTRCD. LVEF <55% on routine surveillance during HER2-targeted therapy indicates a risk of HF

**Table 4:** Gaps in the literature.

1	Lack of standardization in the use of imaging modalities for cardiotoxicity assessment and in the protocol used during image acquisition.
2	There is a need for studies that evaluate the utility of multimodal imaging approaches for cardiotoxicity assessment.
3	Lack of standardization in the interpretation of imaging findings.
4	Lack of research on the cost-effectiveness of different imaging modalities for cardiotoxicity assessment.
5	Insufficient research into the long-term prognostic value of imaging modalities in assessing cardiotoxicity.
6	Evaluation of the utility of multimodality imaging approaches for cardiotoxicity assessment.

## Data Availability

All data is available upon request.
